# EEG-Based Analysis of Neural Responses to Sweeteners: Effects of Type and Concentration

**DOI:** 10.3390/foods14142460

**Published:** 2025-07-14

**Authors:** Xiaolei Wang, Guangnan Wang, Donghong Liu

**Affiliations:** 1 College of Biosystems Engineering and Food Science, Zhejiang University, Hangzhou 310058, China; a2214354318@163.com (X.W.); 22113059@zju.edu.cn (G.W.); 2 College of Biosystems Engineering and Food Science, Beijing Technology and Business University, Beijing 100048, China

**Keywords:** scalp electroencephalogram, non-nutritive sweeteners, sweeteners, brain response, taste

## Abstract

Sweetness is a key dimension of sensory experience in food, and variations in the type and concentration of sweeteners can elicit distinct brain responses. In this study, electroencephalography (EEG) was employed to systematically evaluate neural activity elicited by different concentrations of sucrose solutions (1%, 3%, 5%, and 7%) and by non-nutritive sweeteners matched in perceived sweetness to a 7% sucrose solution (10% erythritol, 0.0133% sucralose, and 0.0368% stevioside). The results revealed that an increased sucrose concentration was associated with progressively weaker EEG signal intensity, suggesting that the brain can effectively distinguish sweetness intensity. Under iso-sweet conditions, different types of sweeteners induced significantly distinct EEG patterns, indicating that the nature of the sweetener modulates flavor perception at the neural level. Further analysis showed increases in both δ- and α-band power following sweet taste stimulation, with prominent activations observed in the frontal, parietal, and right temporal regions. These findings demonstrate the utility of EEG in detecting subtle differences in brain responses to sweeteners, offering new insights into the neural mechanisms underlying sweet taste perception.

## 1. Introduction

Sweeteners, as pleasurable food additives, have garnered widespread attention worldwide in recent years. Although consumer preference for sweetener-based products is often attributed to their “low-sugar” or “sugar-free” health benefits, it is undeniable that the sensory perception of sweeteners themselves plays a crucial role in influencing consumer choices [1]. Current research primarily focuses on the effects of sweeteners on metabolic health and appetite regulation; however, the specific mechanisms by which different types and concentrations of sweeteners influence taste, flavor, and mouthfeel perception remain insufficiently explored [2]. The type and dosage of sweeteners are key factors affecting sensory attributes. Due to differences in molecular structure and sweetness thresholds, various sweeteners may present diverse sensory characteristics such as sweetness intensity, an aftertaste, bitterness, or metallic notes [3]. Therefore, to comprehensively understand the role of sweeteners in flavor construction, it is essential to clarify the perceptual differences associated with different types and concentrations.

The consumption of food and beverages containing sweeteners is a multisensory experience, and flavor perception is closely related to conscious processes, which can be categorized into explicit and implicit perception. As a result, researchers typically adopt different measurement methods based on study objectives, classifying approaches into explicit and implicit measurements. Explicit measures involve participants’ active self-reporting after tasting, whereas implicit measures rely on continuous physiological recordings, such as brain activity, to capture real-time responses during tasting [4]. Although explicit measurements are convenient for data collection and analysis, reliance on subjective evaluations alone often results in large inter-individual variability, which can obscure subtle differences in flavor perception. Therefore, introducing implicit measurement techniques can help unravel the complex mechanisms underlying taste perception.

Physiologically, the brain plays a pivotal role in processing emotional responses and preferences related to food flavor. Especially when participants are unable to clearly articulate their perceptions, recording brain activity becomes a valuable tool for assessing changes in flavor perception. However, the application of implicit measures in food science to study flavor perception remains relatively limited. Therefore, this study specifically investigates the applicability of EEG as an implicit measurement tool in the context of flavor perception and presents several preliminary findings supporting its effectiveness.

During the tasting of sweetener-containing samples, gustatory information undergoes a series of complex neurocomputational processes in the brain and is ultimately transformed into specific sensory attributes. This process generates distinct temporal dynamics across different brain regions. Thus, a neuro-monitoring method with high temporal resolution is required to accurately capture the flavor stimulation process. Non-invasive techniques commonly used to monitor brain activity include electroencephalography (EEG), functional near-infrared spectroscopy (fNIRS), and functional magnetic resonance imaging (fMRI). Among these, EEG stands out due to its millisecond-level temporal resolution, allowing for the rapid detection of brain responses under experimental conditions [5]. Furthermore, EEG devices are portable, making them suitable for brain imaging studies in real-world settings beyond clinical or laboratory environments. EEG enables the recording of electrophysiological indicators that reflect the overall cortical neuronal activity in response to different flavor stimuli.

Han et al. employed EEG to investigate brain responses to equally sweet stimuli (sucrose, aspartame, and their mixtures) [5]. Despite participants’ inability to distinguish the sweeteners consciously, the EEG results revealed significantly different cortical activations [6,7]. Wu et al. further demonstrated that high-intensity-taste stimuli can induce variations in brain oscillatory patterns, with alpha and theta frequency bands exhibiting high sensitivity to different taste qualities [8]. Additionally, source localization analyses have indicated that sweet-induced brain activity is associated with regions involved in reward processing, emotional eating, and motivational tendencies [9]. Wu et al. utilized EEG to study the brain’s perception of three different umami compounds and found that such stimulation enhanced alpha-wave activity in the frontal, parietal, and occipital brain regions [8]. These studies highlight the considerable potential of EEG in decoding brain activity elicited by taste stimuli. Integrating EEG-based implicit indicators with traditional explicit evaluations can thus provide deeper insight into the neural processing of sweetener-related flavor experiences.

Nevertheless, there is still a lack of systematic research exploring how variations in sweetener types and concentrations affect brain physiological signals. Given the differing sweetness intensities and flavor characteristics among various sweeteners (e.g., sucrose, erythritol, sucralose, and stevioside), it is of great significance to elucidate how these differences modulate brain neural responses to better understand the underlying mechanisms of flavor perception.

This study aims to investigate the following three aspects:

(1) Monitoring of brain physiological signals induced by different types and concentrations of sweeteners using EEG;
(2) Analysis of the regional brain activity and neural oscillations in response to various sweetener stimuli;
(3) Exploration of the correlations between sweetener stimuli and changes in EEG-derived physiological signals.

Through implicit measurements, this study seeks to evaluate how different sweeteners shape brain responses, offering novel perspectives and theoretical support for understanding the neural mechanisms and perceptual construction of sweet taste.

## 2. Materials and Methods

### 2.1. Chemicals

Food-grade sucrose, erythritol, sucralose, and stevioside were all obtained from Qingdao Fuyuan Biotechnology Co., Ltd. (Qingdao, Shandong, China). All reagents used were of food-grade quality. Ultrapure water was prepared using an NW10VF purification system (Heal Force Development, Ltd., Hong Kong SAR, China).

### 2.2. Taste Samples

To investigate the effects of sweetener type and concentration on brain responses, seven taste stimuli were prepared. These included sucrose solutions at four concentrations (1%, 3%, 5%, and 7%) to evaluate the effect of sweetness intensity, and three iso-sweet non-nutritive sweeteners (10% erythritol, 0.0133% sucralose, and 0.0368% stevioside) matched to the sweetness of 7% sucrose. Ultrapure water was used as the control sample.

### 2.3. Participants

The study protocol was approved by the Ethics Review Committee of Zhejiang University (approval no. 2024-2 and approved on 16 January 2024), and all procedures complied with the Declaration of Helsinki. A total of 30 right-handed healthy Chinese participants (15 males and 15 females), aged between 18 and 30 years, were recruited. All participants were university students who were familiar with the taste of sweeteners, reported no allergies to the tested compounds, and had no history of smoking or alcohol consumption. Participants were asked to refrain from eating, drinking (except water), and smoking for 2 h before testing. All EEG tests were conducted in a quiet, temperature-controlled (21 ± 1 °C), and neutral-smelling EEG laboratory. Written informed consent was obtained from all participants, who were compensated financially for their participation.

### 2.4. EEG Recording

EEG data were recorded using the Curry 9 system with a 64-channel Quik-Cap (Compumedics Europe GmbH, Freiberg, Germany) connected to a SynAmps 2/RT amplifier. The reference electrode was placed at FCz. Impedance was maintained below 5 kΩ, and data were sampled at 1000 Hz. The amplifier was configured in DC mode, with a low-pass filter set at 400 Hz and a resolution of 24 nV/LSB.

Prior to the formal EEG task, pre-tests were conducted to assess resting-state and emotion-related signals under conditions including eyes open/closed, facial expressions, and internal reflection. Participants were trained to remain still, minimize facial movements, and keep their eyes closed unless otherwise instructed.

### 2.5. Procedure of the Formal EEG Experiment

After putting on the EEG cap, participants were instructed to relax. Each trial began with a 30 s mouth rinse using ultrapure water. Subsequently, seven taste stimuli (including water, four concentrations of sucrose, and three non-nutritive sweeteners) were presented in order of increasing sweetness concentration. Each sample was provided in a 5 mL aliquot. Participants were instructed not to swallow the sample or move their heads during tasting to minimize motion artifacts.

Each trial followed the procedure below:

- The experimenter gave a “start” cue, which was simultaneously marked as “0” in the EEG system, followed by a 10 s resting period.
- The stimulus sample was placed in the participant’s mouth, marked as “1”.
- After the sample was held in the mouth for 5 s, it was marked as “2”; the participant then spat out the liquid and rinsed their mouth.
- A 60 s rest period followed to allow EEG signals to return to baseline before the next trial.

**Figure 1:**
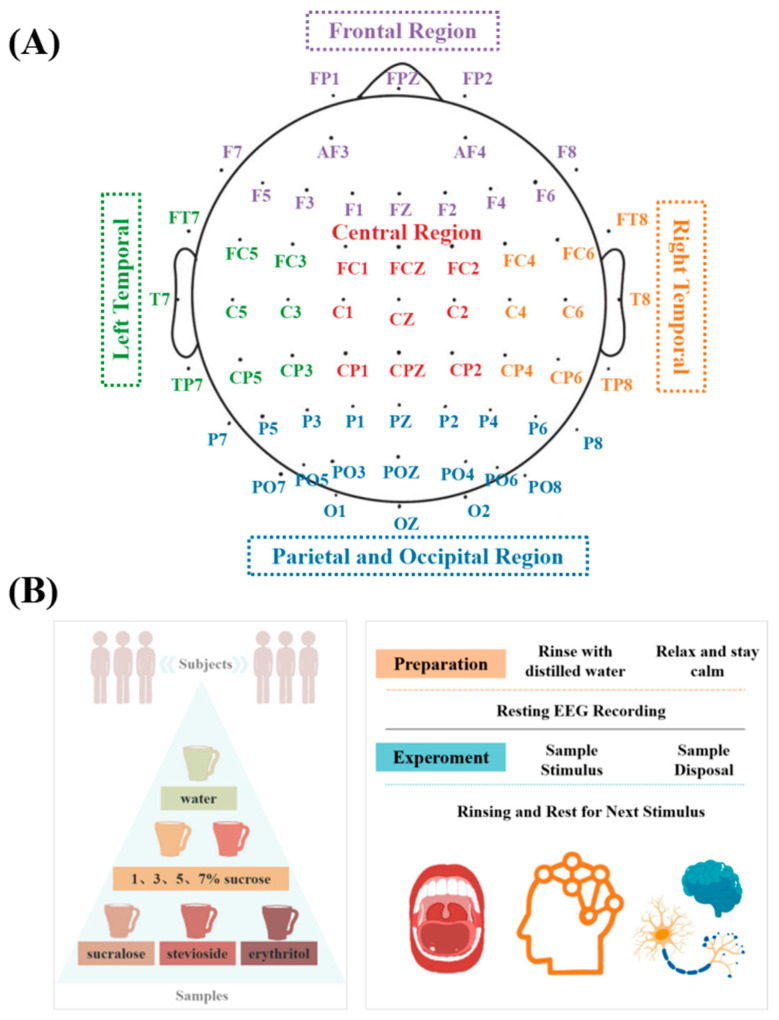
(**A**) The distribution of 64 EEG electrodes and their division into different brain regions according to the 10–20 international system. (**B**) A schematic diagram of the EEG signal acquisition process under different sample stimulations.

Each stimulus was tested three times, and EEG signals were recorded continuously throughout the entire experiment (Figure 1B).

### 2.6. EEG Pre-Processing and Analysis

Raw EEG signals were processed in MATLAB v2023.a (The MathWorks, Natick, MA, USA), including channel alignment, filtering, epoching, artifact rejection, ICA, and re-referencing. Data from participants with excessive noise were excluded (~15%). A 2–3 s stable segment was selected for analysis based on recommendations in the literature [10].

Power spectral density (PSD) was calculated using MNE-Python 1.9.0 and custom Python scripts (Welch’s method, Hamming window, 1.95 Hz resolution, 10% overlap). For each stimulus condition, EEG trials were repeated three times per subject, and the resulting power spectral densities were averaged across repetitions before computing the AUC for each frequency band. PSD areas (AUC) were computed for delta (1–4 Hz), theta (4–8 Hz), alpha (8–13 Hz), and beta (13–30 Hz) bands. EEG data were analyzed regionally across six brain zones: frontal (FR), left temporal (LT), right temporal (RT), central (CR), parietal–occipital (PO), and global average (All) (Figure 1A). AUC values were averaged across channels and are expressed in μV^2^/Hz.

A mixed-model ANOVA with Bonferroni post hoc comparison was used to assess differences across stimuli, brain regions, and frequency bands. Stimuli, brain regions, and frequency bands were treated as fixed factors, and participants as random factors. Spearman correlation was used to examine the relationship between stimulus type and EEG signal changes.

### 2.7. Statistical Analysis

Statistical tests (ANOVA and Spearman correlation) were performed in IBM SPSS Statistics (v17.0, Chicago, IL, USA). Significance was set at *p* < 0.05. All figures were generated using GraphPad Prism (v14, GraphPad Software, San Diego, CA, USA).

## 3. Results

### 3.1. ANOVA of Brain Response to Different Sweeteners

A total of 30 healthy right-handed participants were initially recruited for the EEG experiment. However, data from two male participants were excluded due to excessive ocular and muscular artifacts during the resting-state baseline period, likely caused by frequent eye movements or other involuntary body motion while their eyes were closed [11]. Consequently, EEG data from 28 participants were included in the final analysis. To reduce inter-subject variability and minimize systematic error, all EEG data were baseline-normalized using the pre-stimulus resting state.

**Figure 2:**
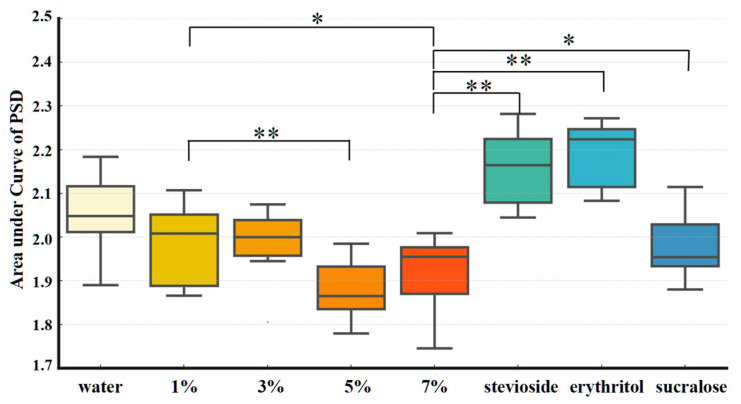
Differential variance analysis of overall brain signal responses elicited by 8 stimuli (water, 1%, 3%, 5%, and 7% sucrose solutions, and three non-nutritive sweeteners). * *p* < 0.05, ** *p* < 0.01.

The brain’s response to each sweetener stimulus was quantified using the area under the curve (AUC) of power spectral density (PSD) within the 1–30 Hz frequency range (Figure 2) [12]. The analysis revealed a decreasing trend in EEG signal strength with increasing sucrose concentration: the AUC values at 5% and 7% sucrose were significantly lower than those at 1% (*p* < 0.05). This pattern suggests potential neural adaptation or sensory saturation effects in response to higher concentrations of sucrose [13].

Notably, under iso-sweetness conditions (matched to the perceived sweetness of 7% sucrose), the three non-nutritive sweeteners-stevioside, erythritol, and sucralose-elicited markedly stronger neural responses. Among them, stevioside elicited the strongest EEG response, followed by erythritol, while sucralose also induced significantly higher activation than 7% sucrose. These findings suggest that the brain can differentiate not only between varying levels of sweetness but also between sweeteners of different chemical compositions, even when they evoke similar taste intensities [13].

To statistically assess these effects, a mixed-effects ANOVA was conducted, followed by Bonferroni post hoc pairwise comparisons, outlined below.

Sucrose concentration: Significant differences were observed between sucrose concentrations. The AUC values at both 5% (*p* = 0.0002, **) and 7% (*p* = 0.0024, *) sucrose were significantly lower than that at 1%.

Sweetener type: All three non-nutritive sweeteners induced significantly stronger EEG responses than 7% sucrose. Erythritol and stevioside showed highly significant differences (*p* < 0.001), while sucralose also differed statistically (*p* = 0.037) [14].

In summary, these results indicate that the brain’s responses are modulated not only by sweetness intensity but also by sweetener type, likely reflecting differences in molecular structure, receptor activation, and associated sensory or emotional processing.

### 3.2. Brain Rhythm Responses to Sweetener Stimuli

**Figure 3:**
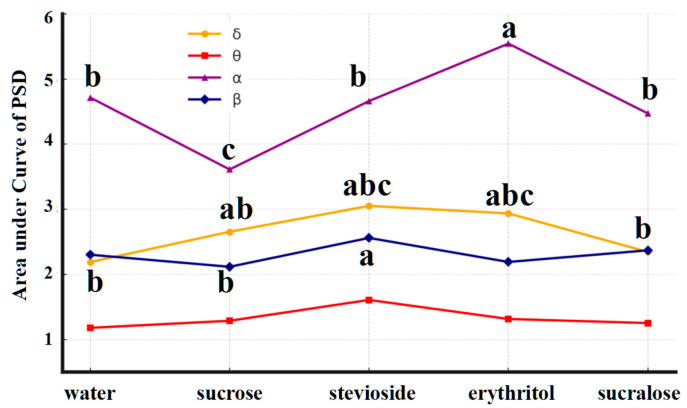
Brain rhythm wave responses elicited by 5 stimuli (water, 7% sucrose solution, and three non-nutritive sweeteners). a, b, c present statistically significant differences (*p* < 0.05).

To further investigate the neural oscillatory characteristics elicited by sweet taste stimulation, the present study computed the area under the curve (AUC) of power spectral density (PSD) across four canonical frequency bands-delta (δ, 1–4 Hz), theta (θ, 4–8 Hz), alpha (α, 8–13 Hz), and beta (β, 13–30 Hz) (Figure 3)-aiming to explore the differential modulation of brain rhythm activity by various sweeteners.

The results revealed that under all stimulus conditions, alpha-band activity was consistently higher than that of other frequency bands. This pattern likely reflects an intrinsic physiological rhythm, as alpha waves are naturally dominant in resting-state EEG when individuals are awake, are relaxed, and have their eyes closed [15,16,17]. However, this baseline feature did not hinder the comparative evaluation of changes in alpha power across different sweeteners. Notably, the non-nutritive sweeteners-particularly stevioside and erythritol-induced stronger EEG power responses across most frequency bands compared to sucrose and water [13]. For example,

In the delta band, both stevioside and erythritol elicited significantly higher AUC values than water and sucrose.

In the alpha band, erythritol also induced greater activity than sucrose.

Although sucralose exhibited relatively weaker responses in the delta band, it still outperformed sucrose in the beta band.

These findings suggest that different types of sweeteners not only vary in the magnitude of brain responses but also exhibit distinct frequency-specific neuromodulatory patterns [18]. Among them, stevioside consistently evoked the strongest and most-stable responses in both the alpha and delta bands, which are commonly associated with emotional regulation, attentional processing, and gustatory integration [19].

Although the current study did not conduct full statistical testing across all frequency bands, the preliminary findings indicate that non-nutritive sweeteners, under iso-sweetness conditions, may activate broader or more robust cortical responses than sucrose. This implies that their neural processing may involve enhanced sensory and affective dimensions of flavor perception. Overall, these results further demonstrate the utility and sensitivity of EEG as a tool for decoding brain responses related to flavor perception and emotional experience.

### 3.3. PSD of Brain Response to Different Sweeteners

**Figure 4:**
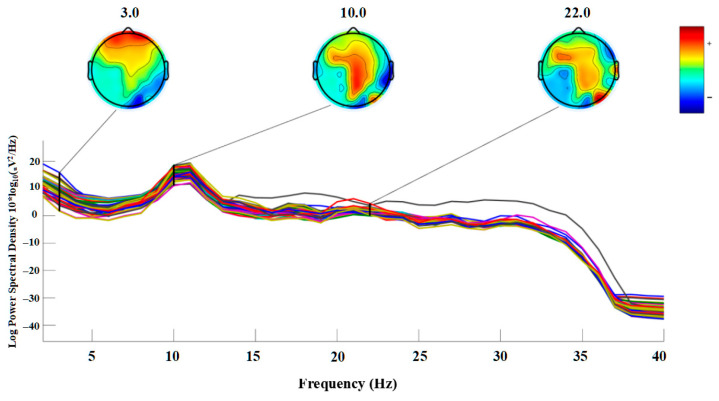
PSD results of brain response to 7% sucrose solution between 1 and 30 Hz. The different colored lines represent EEG activity from distinct brain regions, with colors matched to those in Figure 1A.

Figure 4 presents an example of power spectral density (PSD) activity recorded from a randomly selected participant during the tasting of a 7% sucrose solution. The results show clear EEG responses across all channels within the 1–30 Hz range, with the most prominent signal centered around approximately 10 Hz. This frequency-specific response pattern was highly consistent among other participants. Moreover, based on previous experimental findings, similar PSD patterns have also been observed under other types of stimuli such as alcohol and umami peptides.

These findings suggest that this PSD pattern likely reflects the participant’s experimental state-specifically, eyes-closed and resting conditions-rather than being a sample-specific neural response [20]. However, many EEG studies on taste perception have failed to clarify this, often assuming that such PSD patterns directly reflect the characteristics of the taste stimuli. This highlights the importance of detailed PSD analysis in EEG-based flavor perception research. In the present study, PSD was primarily analyzed in terms of spectral power across conventional frequency bands. However, future work may consider more advanced PSD analysis methods, such as time–frequency decomposition, connectivity measures, or source-level spectral mapping, to capture the full complexity of neural dynamics involved in taste perception.

### 3.4. Time-Domain Analysis of Brain Responses to Different Sweet Taste Stimuli

**Figure 5:**
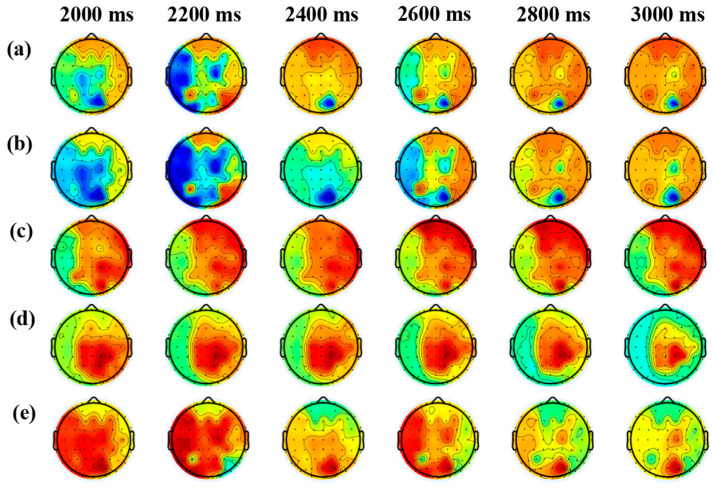
Grand average brain response of one randomly selected subject to 5 stimuli: (**a**) water, (**b**) 7% sucrose, (**c**) stevioside, (**d**) erythritol, and (**e**) sucralose. Color coding is relative to the absolute maximum potential difference. The more towards red indicates higher signal strength, while the more towards blue indicates weaker signal strength.

To further elucidate the temporal dynamics of neural responses induced by sweet taste perception, time-resolved topographic EEG maps were analyzed within the key gustatory processing window (2000–3000 ms). Figure 5 illustrates representative cortical activation patterns elicited by different sweeteners over time in a randomly selected participant.

The results revealed that the brain’s response to non-nutritive sweeteners emerged around 2000 ms. Among them, stevioside induced the most robust and widespread cortical activation, with signal strength progressively increasing throughout the 2000–3000 ms interval. In contrast, although erythritol and sucralose also triggered noticeable responses beginning at approximately 2000 ms, their activation levels peaked by 2600 ms and declined rapidly thereafter. This suggests that these sweeteners elicited shorter-lasting neural effects, with signals dissipating soon after the initial stimulus onset. The activated regions for all three non-nutritive sweeteners were predominantly located in the frontal (FR) and right temporal (RT) cortices, areas closely associated with emotion regulation and taste processing.

In comparison, sucrose evoked weaker responses with slower onset and limited spatial distribution. Notably, stevioside consistently induced the earliest, strongest, and most spatially extensive EEG responses across multiple time points. This supports the notion that stevioside may engage faster and more-prominent neural encoding mechanisms during sweet taste perception. These observations are in line with prior frequency-domain analyses, reinforcing the superior EEG responsiveness of non-nutritive sweeteners-particularly stevioside and erythritol-compared to traditional caloric sweeteners.

In summary, time-domain analyses indicate distinct neural processing profiles for different sweeteners, with marked differences in both response intensity and spatial patterns over time. These findings highlight the specificity and complexity of brain responses to chemically diverse sweet taste stimuli.

### 3.5. Regional Brain Response Differences to Sweeteners

**Figure 6:**
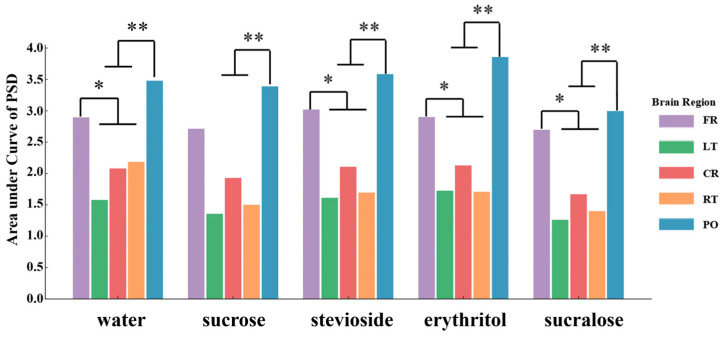
ANOVA of different brain region responses to 5 stimuli (water, 7%, and three no-sugar sweeteners). * *p* < 0.05, ** *p* < 0.01.

As illustrated in Figure 6, sweetener stimuli elicited varied EEG signal responses across different brain regions. Among all samples, the parietal–occipital (PO) region exhibited consistently stronger PSD responses compared to other regions [21]. This suggests that the PO region may play a dominant role in processing taste perception. The frontal region (FR) also showed relatively high activation, particularly in response to erythritol and stevioside, indicating its potential involvement in flavor-related attentional or emotional processing [22].

Notably, non-nutritive sweeteners such as stevioside and erythritol induced significantly higher signal intensities in both the PO and FR regions compared to sucrose, which exhibited more moderate and spatially restricted responses. This highlights that different sweeteners modulate cortical activity in a region-specific manner, likely reflecting both sensory discrimination and hedonic processing mechanisms.

These results emphasize the capacity of EEG to detect spatially distinct brain responses to sweet stimuli and support its application in evaluating the neurocognitive impact of food-related sensory experiences.

## 4. Discussion

This study employed scalp electroencephalography (EEG) to investigate the neural processing of different sweeteners, focusing on both sweetness intensity (1% to 7% sucrose) and sweetener type (stevioside, erythritol, and sucralose). The findings revealed that the brain’s response is not only sensitive to the concentrations of sweeteners but also discriminative toward different sweetener types, even when matched for perceived sweetness.

The results demonstrated that higher concentrations of sucrose (5% and 7%) elicited significantly lower EEG responses compared to 1% sucrose (*p* = 0.0002 and *p* = 0.0024, respectively), suggesting potential neural adaptation or sensory saturation. In contrast, non-nutritive sweeteners evoked stronger EEG responses than 7% sucrose across most conditions. Specifically, both stevioside and erythritol showed highly significant increases (*p* < 0.001), while sucralose also induced a statistically significant enhancement (*p* = 0.037), indicating the brain’s heightened sensitivity to their chemical properties.

In the frequency domain, non-nutritive sweeteners elicited significantly stronger EEG activity than sucrose, particularly in the delta (1–4 Hz) and alpha (8–13 Hz) bands. These frequency bands are commonly associated with emotional regulation, attention, and gustatory integration. Alpha-band activity was the most dominant across all conditions, reflecting both resting-state characteristics and sweetener-specific modulation. Among the sweeteners, stevioside consistently evoked the strongest and most-stable neural responses, supporting its unique neurosensory impact.

Time-domain analysis further supported these findings. Stevioside elicited the earliest and most-widespread cortical activation within the 2000–3000 ms post-stimulation interval, whereas erythritol and sucralose induced shorter-lived responses that declined after 2600 ms. In contrast, sucrose produced delayed and relatively weaker activation patterns. The frontal (FR) and right temporal (RT) lobes showed prominent activation for non-nutritive sweeteners, suggesting involvement in emotion and taste processing.

Additionally, region-specific analysis revealed that the parietal–occipital (PO) region exhibited consistently higher signal amplitudes across all conditions. While water stimulation also produced activity in this area, the spatial distribution and magnitude differed across stimuli. The elevated responses in the PO and FR regions in response to sweeteners such as stevioside and erythritol may reflect distinct pathways of sensory and hedonic processing [21,22].

Taken together, these findings indicate that different sweeteners evoke distinct spatiotemporal patterns of neural activity. The differences are likely driven not only by perceived intensity but also by molecular structure and receptor activation. These insights may inform future research on consumer preferences and the design of healthier sugar alternatives that preserve desirable sensory experiences.

Moreover, this study underscores the utility of EEG as a sensitive and objective tool for decoding flavor perception. Unlike subjective self-reporting, EEG provides real-time and high-resolution tracking of cortical responses, making it a powerful method for exploring implicit sensory and emotional experiences. This approach may contribute to more ecologically valid assessments in food and nutrition science.

## 5. Conclusions

This study demonstrated that different concentrations and types of sweeteners elicit distinct neural responses, as assessed by EEG-based spectral and temporal analyses. Sucrose at higher concentrations showed a decreasing trend in EEG signal intensity, suggesting potential neural adaptation or sensory saturation. In contrast, non-nutritive sweeteners-particularly stevioside and erythritol-induced stronger and more-persistent brain responses, especially in regions related to gustatory and emotional processing.

These findings highlight the brain’s capacity to differentiate sweeteners based on both intensity and chemical nature, even under iso-sweetness conditions. Furthermore, the integration of frequency- and time-domain EEG analyses provides a comprehensive view of how sweet taste is encoded in the brain. This research offers new insights into the neurophysiological mechanisms underlying sweet taste perception and may contribute to the development of more effective sugar alternatives in the food industry.

## Data Availability

The data presented in this study are available on request from the corresponding author due to privacy or institutional restrictions.

## Abbreviations

The following abbreviations are used in this manuscript:

EEG	Electroencephalography
PSD	Power spectral density
AUC	Area under the curve
FR	Frontal region
LT	Left temporal region
CR	Central region
RT	Right temporal region
PO	Parietal–occipital region
